# Microbial electrochemical technologies: maturing but not mature

**DOI:** 10.1111/1751-7915.13045

**Published:** 2017-12-26

**Authors:** Federico Aulenta, Sebastià Puig, Falk Harnisch

**Affiliations:** ^1^ Water Research Institute (IRSA) National Research Council (CNR) via Salaria km 29,300 00015 Monterotondo RM Italy; ^2^ Laboratory of Chemical and Environmental Engineering (LEQUiA) Institute of the Environment University of Girona Carrer Maria Aurèlia Capmany 69 17003 Girona Spain; ^3^ Department of Environmental Microbiology Helmholtz‐Centre for Environmental Research GmbH – UFZ Permoserstraße 15 04318 Leipzig Germany

It started more than 100 years ago (Potter, 1911). As most, if not all, important developments, its discovery was triggered by mere curiosity. Obviously, what we are referring to is a field of science and engineering that is now summarized under the umbrella of microbial electrochemical technologies (MET). MET are based on the interfacing of microbiology and electrochemistry. Primary MET are based on the wiring of microbial metabolism to solid‐state electrodes, via a process typically referred to as extracellular electron transfer (EET). Until recently, EET remained a scientific curiosity. However, advances in the physiology, phylogeny and even biochemistry of so‐called ‘electroactive’ bacteria have gained deeper insights and shown that electroactives are more abundant and important than considered, so far. This research was also driven by interest in low environmental impact biotechnologies and has created a staggering array of processes including bioproduction, bioremediation, wastewater treatment, biosensing and many more. Key to all of these MET‐based bioprocesses is the promise to be more sustainable from an environmental standpoint while ensuring an unpreceded control over microbial reactions.

Today, research on MET is highly represented by a young and dynamic scientific society that organized itself amongst other in the International Society for Microbial Electrochemistry and Technology (ISMET). ISMET is pooling researchers from various areas of science and engineering, spanning from microbiology and electrochemistry to chemical engineering and material science.

Notably, the progress in the development and application of MET we witnessed within the last decade was largely boosted by improvements in the available tools and infrastructures, as well as by important societal changes. For instance, affordable high‐throughput sequencing that could hardly be imagined only a few years ago is now on our daily agenda, and the general awareness that electric power (from renewables) is a resource that should be used to drive (bio)chemical synthesis is almost common sense. Therefore, MET could contribute driving the transition to a stronger and more circular economy where resources are used in a more sustainable way.

However, despite ever‐increasing research efforts being in line with industry, and government interest worldwide, processes and devices based on microbial electron transfer are not yet available on the market. Regardless their final application, this is mostly because of their low conversion efficiency, limited reliability and complex scalability. These are some of the main scientific and technical challenges that papers included in this special issue of *Microbial Biotechnology* highlight. Hopefully, this special issue will represent an important contribution in the field and will help driving the transition of MET out of the laboratory, all the way to the market.

This special issue *Microbial Electrochemical Technologies coming of Age* of *Microbial Biotechnology* comprises eleven articles (one highlight, two reviews, one brief report and seven research articles). These contributions span thematically from microbial ecology to environmental biotechnology.

Arends (2017) highlights the recent development of encapsulated microbial catalysts to be used for applications and research questions that need reproducibly coated microbial bioelectrodes or fast responses. In his highlight, special attention was given to the recent work of Estevez‐Canales et al. (2017) considering that it makes a step forward in prolonging the shelf life and optimizing storage conditions of precolonized bioanodes.

Microbial ecology provides an arsenal of techniques targeting different phylogenetic and functional levels. However, it is not always clear which methodical approach is suited best to answer a specific research question properly. To reduce this gap, the review of Koch et al. (2017) provides insights into the relevance of microbial ecology for the characterization as well as future engineering and management of microbial electrochemical technologies. In line, Malanoski et al (2017) provide guidance for using metagenomic and 16S‐based sequencing to characterize low complexity microbial communities that may contain previously uncharacterized microorganisms, such as those associated with biocathodes. Lewis et al. (2017) provide a foundation from which to build on for understanding biocomplexity in bioelectrochemical systems for conversion of biomass‐derived streams and towards the development of community management and engineering strategies for enabling renewable hydrogen production.

Lusk et al. (2017) employed an enriched mixed culture of thermophilic bacteria for the purpose of showing that higher current densities from cellulose are possible in thermophilic MET. A considerable enhancement of current output could also be achieved using quorum sensing activation using ferrous iron or sulphur as electron donors that remarkably increased electrode colonization by *Acidithiobacillus ferrooxidans* (Chabert *et al*., 2017). Microbial population in biocathodes could also influence the global metabolism in mixed culture fermentation. Here, with their recent work published in the issue, Moscoviz et al. (2017) proved that the electro‐fermenting microbial community was more efficient for producing 1,3‐propanediol when compared with fermentation controls

This special issue also contributes in exploring various configurations of biocathodes and bioanodes for bioremediation applications. Pous et al. (2017) state that microbial electro‐remediation represents a unique opportunity to develop a robust, resilient and sustainable technology in a circular economy context to deal with different contaminants that are already present in our groundwater bodies and soils. Two research articles are aligned with this view. Palma et al. (2017) describe a bioelectrochemical reactor configuration, the ‘bioelectric well’, which can be installed directly within groundwater wells and that is to be applied for in situ treatment of organic contaminants, such as petroleum hydrocarbons. Domínguez‐Garay *et al*. (2017) developed a so‐called bioelectroventing strategy for achieving an effective clean‐up of the atrazine‐polluted soils able to restore the prepollution conditions.

In summary, these articles –that can only provide a snapshot of the current rapid and significant advancement in the field –illustrate that MET are maturing. This could also be witnessed on the recent worldwide conferences in Rome (EU‐ISMET 2016) as well as its worldwide pendant in Lisbon (ISMET 6, 2017). We are already curious to see what will be the significant progress as well as the new kid on the block in 2018 –to be seen, for instance, in Newcastle at the EU‐ISMET 2018.

## Conflict of interest

None declared.
